# The role of agomelatine in appetite regulation and body weight in rats

**DOI:** 10.1113/EP092783

**Published:** 2025-08-13

**Authors:** Engin Korkmaz, Yavuz Erden, Çiğdem Tekin, Suat Tekin

**Affiliations:** ^1^ Faculty of Medicine, Department of Physiology İnönü University Malatya Türkiye; ^2^ Faculty of Science, Department of Molecular Biology and Genetics Bartın University Bartın Türkiye; ^3^ Vocational School of Health Services, Department of Health Care Services İnönü University Malatya Türkiye

**Keywords:** agomelatine, appetite, food intake

## Abstract

The hypothalamic nuclei play a central role in the synthesis of anorexigenic and orexigenic neuropeptides, which are regulated by peripheral hormones, like leptin and ghrelin. Melatonergic receptors (MT_1_/MT_2_) are prominently expressed in the arcuate nucleus of the hypothalamus – an essential hub for appetite control – and in peripheral metabolic tissues where leptin and ghrelin are secreted. Agomelatine, an antidepressant drug and potent MT_1_/MT_2_ agonist, offers potential for modulating appetite. This study aimed to investigate the impact of agomelatine on appetite regulation. Forty male Sprague–Dawley rats were randomly allocated into four groups, control (no treatment), vehicle control, agomelatine 20 mg/kg (Ago‐20), and agomelatine 40 mg/kg (Ago‐40), and administered oral gavage for 14 days. Body weight and food intake were recorded daily. At the end of the experiment, rats were euthanized and blood and hypothalamic tissue samples were obtained. Agomelatine significantly reduced body weight (Ago‐40: 275.2 ± 7.2 g vs. control: 339.7 ± 8.3 g, *P <* 0.05) and food intake (Ago‐40: 20.21 ± 1.32 g vs. control: 32.09 ± 1.58 g, *P <* 0.05) by day 14, without affecting water intake. Plasma ghrelin levels decreased (Ago‐40: 22.54 ± 3.95 ng/dL vs. control: 46.67 ± 4.84 ng/dL, *P <* 0.05), while leptin increased (Ago‐40: 552.30 ± 41.67 pg/mL vs. control: 271.10 ± 32.12 pg/mL *P <* 0.05). Hypothalamic orexigenic neuropeptides neuropeptide Y (NPY) and agouti‐related peptide (AgRP) were suppressed (NPY, Ago40: 0.61 ± 0.02 vs. Control: 1.36 ± 0.1321; AgRP, Ago40: 0.52 ± 0.03 vs. Control: 1.49 ± 0.27, *P <* 0.05), while anorexigenic cocaine‐ and amphetamine‐regulated transcript (CART) and proopiomelanocortin (POMC) were elevated (CART: Ago40: 1.19 ± 0.08 vs. Control: 0.92 ± 0.06; POMC: Ago40: 1.49 ± 0.17 vs. Control: 0.67 ± 0.10, *P <* 0.05). These findings suggest agomelatine promotes weight loss by modulating appetite‐related hormones and hypothalamic neuropeptides, highlighting its potential as a therapeutic for obesity and metabolic disorders.

## Abstract (highlights)

- **What is the central question of this study?**
  Does agomelatine, a melatonergic receptor agonist, influence appetite regulation by modulating anorexigenic and orexigenic neuropeptides and peripheral hormones?
- **What is the main finding and its importance?**
  Agomelatine reduced food intake and body weight by increasing anorexigenic proopiomelanocortin/anorexigenic cocaine‐ and amphetamine‐regulated transcript and decreasing orexigenic agouti‐related peptide/neuropeptide Y, alongside lowering ghrelin and elevating leptin. These findings suggest agomelatine may have therapeutic potential for appetite control and weight management.

## 1 INTRODUCTION

Food intake is regulated by a complex physiological system that integrates cognitive and homeostatic signals. In homeostatic regulation, the hypothalamus is a central region controlling feeding behaviour. Specifically, the lateral hypothalamic area acts as the hunger‐regulating centre, while the ventromedial nucleus functions as the satiety‐regulating centre (Ahima & Antwi, 2008). Additionally, the paraventricular, dorsomedial and arcuate nuclei (ARN) of the hypothalamus play critical roles in the central regulation of appetite. In the ARN, neuron groups expressing proopiomelanocortin (POMC), cocaine‐ and amphetamine‐regulated transcript (CART), neuropeptide Y (NPY) and agouti‐related peptide (AgRP) are crucial in regulating energy expenditure and appetite (Simpson et al., 2009). Activation of POMC and CART neurons reduces food intake, while NPY and AgRP neuron activity stimulates feeding (Valassi et al., 2008). Additionally, the hypothalamus integrates signals from peripheral organs with central neuronal inputs to regulate appetite (Han et al., 2022). In this process, leptin secreted by adipose tissue suppresses appetite by enhancing the activation of POMC/CART neurons. In contrast, ghrelin released from the stomach increases the firing rate of NPY/AgRP neurons, thereby stimulating appetite and food intake (Andrews et al., 2008; Varela & Horvath, 2012). Disruptions in these regulatory systems are linked to eating disorders, including anorexia nervosa, bulimia nervosa and obesity (Zigman & Elmquist, 2003). Among these, obesity is the most significant public health challenge, affecting millions worldwide and increasing the risk of serious health problems like type 2 diabetes, cardiovascular disease, cancer and stroke (Pantalone et al., 2017).

Melatonin, secreted by the pineal gland during the dark phase of the light/dark cycle, regulates circadian rhythms and sleep–wake cycles through its MT_1_ and MT_2_ receptors. Beyond its role in circadian rhythm regulation, melatonin has been shown to influence energy expenditure, glucose metabolism and adipogenesis (Suriagandhi & Nachiappan, 2022). Immunoreactivity studies have demonstrated moderate expression levels of MT_1_ and MT_2_ receptors in the ARN (Ng et al., 2017; Wu et al., 2006). These receptors are also involved in regulating leptin secretion from adipose tissue (Alonso‐Vale et al., 2008). Preclinical studies have shown that melatonin activation reduces food intake and exerts weight‐lowering effects by modulating leptin secretion and the activity of NPY/AgRP and POMC neurons (Suriagandhi & Nachiappan, 2022). However, meta‐analyses of clinical studies have reported that melatonin is either ineffective (Loloei et al., 2019; Mostafavi et al., 2017) or has only a mild weight‐reducing effect (Delpino & Figueiredo, 2021). Melatonin's short half‐life and low bioavailability limit its clinical effectiveness (Ahmad et al., 2023). To overcome these limitations, melatonin derivatives targeting melatonergic receptors with improved pharmacokinetic properties have been developed for the treatment of various diseases (Ahmad et al., 2023). Agomelatine, one of these derivatives, has been developed for the treatment of major depressive disorders as a potent agonist at MT_1_ and MT_2_ receptors and a neutral antagonist at serotonergic 5‐HT_2C_ receptors and has been clinically approved in many countries and the European Union (Fasipe, 2019). Considering the roles of melatonergic receptors in the central and peripheral regulation of feeding behaviour, the prolonged half‐life of agomelatine in circulation and its dual receptor effects suggest a potential contribution to appetite regulation. Indeed, studies in obese rodents reported reduced body weight and food intake following agomelatine treatment (Diez‐Echave et al., 2022; Promsan et al., 2022). However, no specific study in the literature has directly investigated the effects of agomelatine on appetite regulation. Based on this background, the present study aims to evaluate agomelatine's effects on appetite regulation by measuring food intake, body weight, key neuropeptides (NPY, POMC, CART, AgRP) and peripheral hormones (leptin and ghrelin) in rats

## 2 METHODS

### 2.1 Ethical approval

The research was approved by the İnönü University Local Ethics Committee for Animal Experiments (2023/3‐1). This study followed the ethical requirements for animal work set by *Experimental Physiology* (Grundy, 2015). The procurement, care and monitoring of the laboratory animals were conducted at the Experimental Animal Production and Research Centre of İnönü University. Experimental analyses were carried out at the Department of Physiology of İnönü University Faculty of Medicine and the Department of Molecular Biology and Genetics at Bartın University Faculty of Science.

### 2.2 Experimental animals and animals and study design

A total of 40 male Sprague–Dawley rats (3 months old) were used in the study. The rats were housed under controlled conditions at 22 ± 2°C with a relative humidity of 50 ± 10% and a 12‐h light–dark cycle. The animals had free access to water and were fed ad libitum with standard rodent chow (Korkutelim Feed Factory, Antalya, Türkiye; 2.8 kcal/g, with 3.2% of the calories derived from fat).

In order to precisely monitor food intake during the experimental period, all animals were housed individually in standard polycarbonate cages (measuring 42 × 26 × 18 cm, standard cages without metabolic cage features). To minimize the stress that could result from individual housing, the animals were acclimated to the cage conditions for 7 days prior to the start of the experiment (Tekin et al., 2017). Feed consumption was determined daily by measuring the amount of feed present in each cage at the beginning of the day and the amount remaining the next day using a precision scale, and calculating the difference. Water intake for each animal was measured using a standard graduated cylinder and recorded daily in millilitres.

Following the acclimatization period, the rats were weighed and randomly assigned to four groups using a computer‐based randomization algorithm (MedCalc 12.7.0 for Windows). One‐way ANOVA revealed no significant differences in body weight among the groups at the start of the experiment (*P* = 0.957). The groups were established as follows:

1. Control group (*n* = 10): no gavage procedure was performed on the rats in this group; only their body weights were recorded.
2. Vehicle group (*n* = 10): hydroxyethylcellulose, the vehicle used for agomelatine, was administered to the rats via oral gavage for 14 consecutive days.
3. Ago‐20 Group (*n* = 10): rats in this group received agomelatine at a dose of 20 mg/kg via oral gavage for 14 consecutive days.
4. Ago‐40 Group (*n* = 10): rats in this group received agomelatine at a dose of 40 mg/kg via oral gavage for 14 consecutive days.

### 2.3 Drug preparation and administration

Agomelatine doses were selected based on prior studies demonstrating its weight loss and appetite‐suppressing effects in obese rats (Promsan et al., 2022). Prescription tablets containing 25 mg of agomelatine (Valdoxan, Servier, Ireland) were ground into a uniform powder using laboratory‐grade mortar and pestle equipment. This powder was mixed with hydroxyethyl cellulose (1 mL/tablet) to prepare an agomelatine suspension. Administered volume was calculated to achieve doses of 20 and 40 mg/kg, based on the body weight of rats.

**FIGURE 1:**
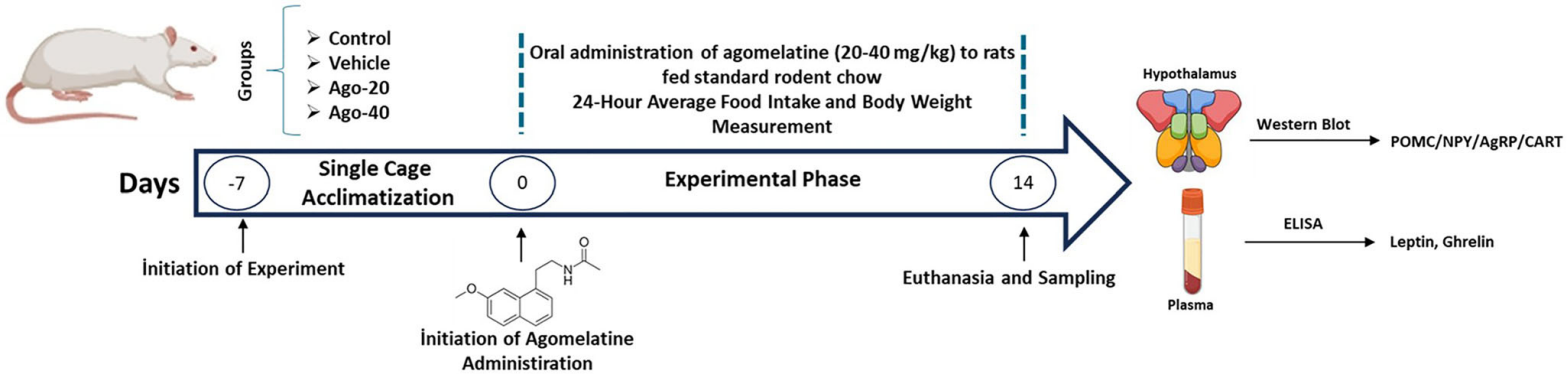
The experimental workflow.

All experimental procedures, including body weight, food intake and water intake measurements, oral gavage and euthanasia, were conducted daily at the same time to maintain consistency. Body weight and food intake measurements were performed prior to agomelatine administration. At the end of the experiment, the animals were euthanized by decapitation, and blood and hypothalamus tissues were obtained. The hypothalamus was localized ventrally by referencing macroscopic anatomical structures (the optic chiasm and infundibulum). This region was carefully isolated through microdissection, immediately frozen on dry ice and stored at −80°C. The experimental workflow is illustrated in Figure 1.

### 2.4 ELISA analyses

Plasma leptin and ghrelin concentrations were quantified using commercially available enzyme‐linked immunosorbent assay (ELISA) kits (Sunred Biotechnology Company, cat. no. 201‐11‐0456 and cat. no. 201‐11‐1650, Shanghai, China) in accordance with the manufacturer's instructions.

### 2.5 Western blot analyses

Hypothalamic tissues were lysed in RIPA buffer using a Bead Mill homogenizer (Tissuelyser LT, Qiagen, Hilden, Germany) (Tekin et al., 2018) Total protein content was quantified using a BCA protein assay kit (Biorbyt cat. no. orb90411, UK). For each sample, 20 µg of protein was separated by 15% SDS‐PAGE and subsequently transferred onto 0.22 µm polyvinylidene difluoride membranes. The membranes were blocked in 5% non‐fat dry milk prepared in Tris‐buffered saline with Tween 20 (TBS‐T) buffer to prevent non‐specific binding. After blocking, the membranes were incubated overnight at 4°C with rat‐specific primary antibodies for POMC (Santa Cruz Biotechnology, Dallas, TX, USA, cat. no. SC‐20148), CART (Santa Cruz Biotechnology, cat. no. SC‐293241), NPY (Santa Cruz Biotechnology, cat. no. SC‐28943), and AgRP (LSBio, cat. no. LS‐C292684, CA, USA), all prepared in 5% BSA. Following primary antibody incubation, the membranes were washed with TBS‐T and incubated for 1 h at room temperature with horseradish peroxidase‐conjugated secondary antibodies (Santa Cruz Biotechnology, cat. no. SC‐516102, Cell Signaling Technology, Danvers, MA, USA, cat. no. 7074) in 5% non‐fat dry milk. The blots were visualized using a chemiluminescent substrate (Clarity ECL Western Blotting Substrate, Bio‐Rad Laboratories, Hercules, CA, USA) and imaged using the Fusion Fx7 imaging system (Vilber Lourmat, Marne‐la‐Vallée, France). Densitometric analysis of the blots was conducted using ImageJ software (Tekin et al., 2019).

### 2.6 Statistical analysis

Statistical analyses were performed using IBM SPSS Statistics software (version 26.0; IBM Corp., Armonk, NY, USA). Quantitative data are presented as the mean ± standard deviation. The normality of data distribution was assessed using the Shapiro–Wilk test. Group comparisons were conducted using the Kruskal–Wallis *H*‐test. When significant differences were detected, *post hoc* multiple comparisons were performed using the Bonferroni‐corrected Mann–Whitney *U*‐test. *P <* 0.05 was considered statistically significant.

## 3 RESULTS

**FIGURE 2:**
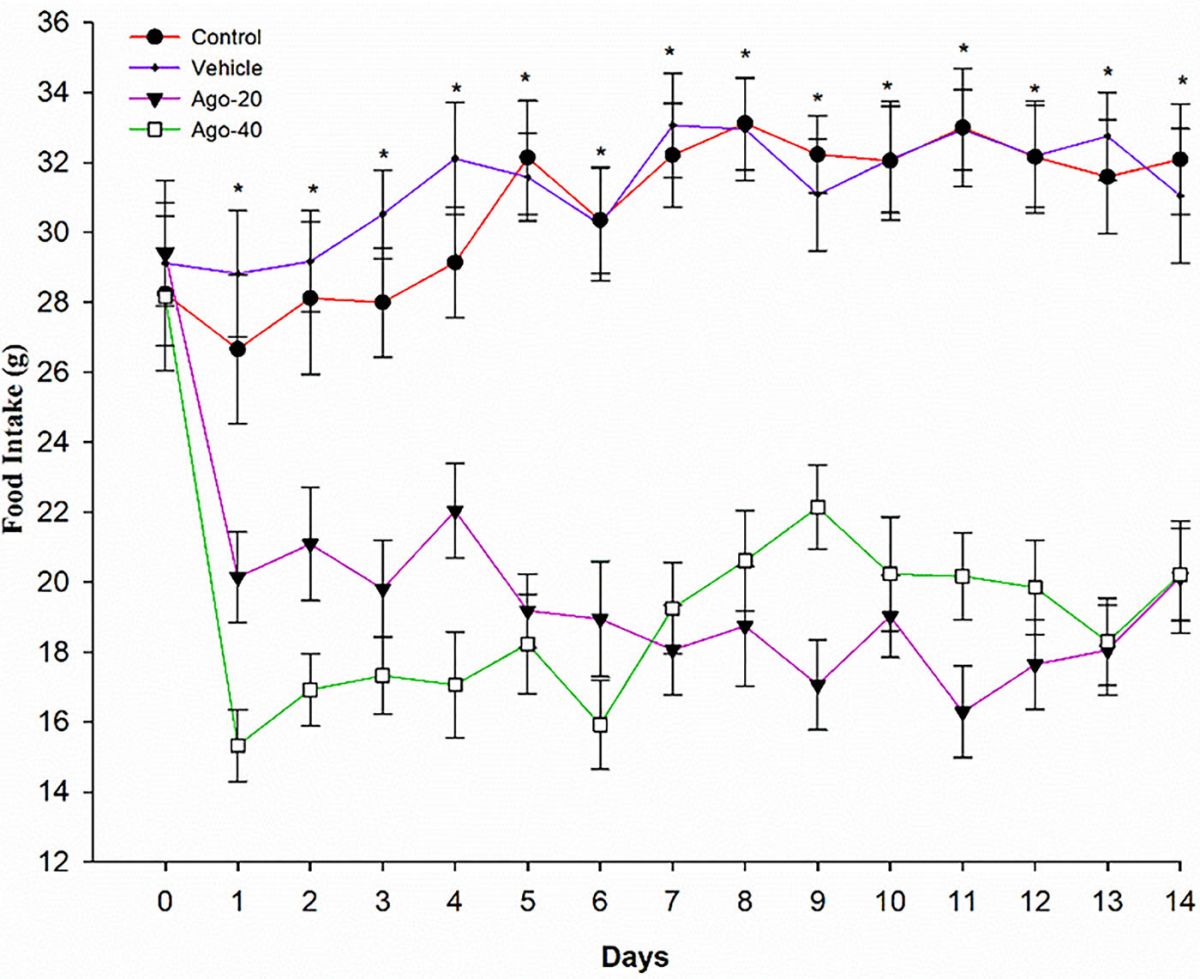
Effects of agomelatine administration on food intake in experimental groups over 14 days. The control group and vehicle group displayed stable food intake throughout the study period, while significant reductions in food intake were observed in both agomelatine‐treated groups starting from day 1 and continuing across all time points, with no significant differences between the Ago‐20 and Ago‐40 groups. Values are expressed as means ± standard deviation. *Statistically significant differences compared to the agomelatine‐treated groups (*P <* 0.05).

**FIGURE 3:**
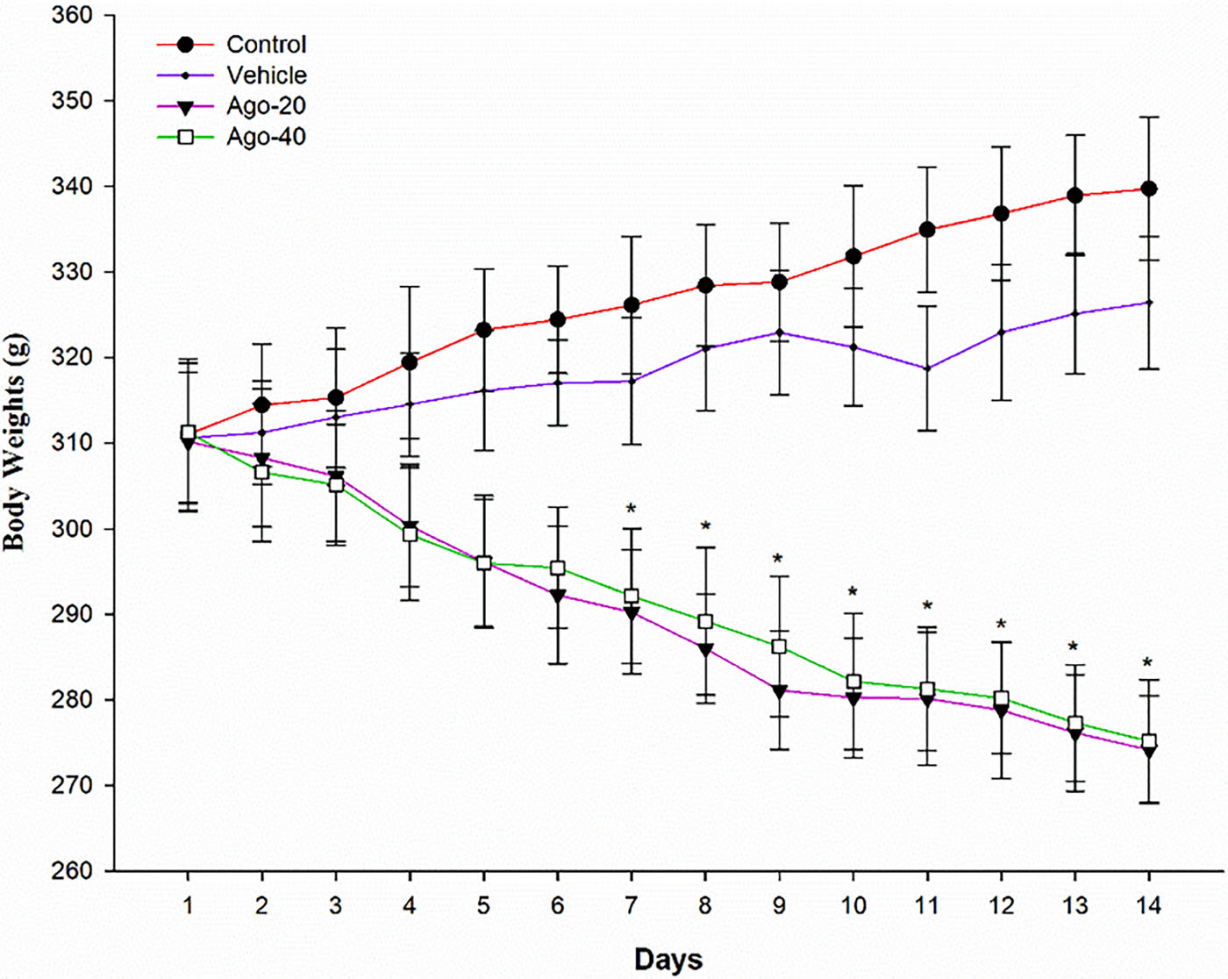
Effects of agomelatine administration on body weight in experimental groups over 14 days. The control group and vehicle group displayed a gradual increase in body weight throughout the study period. In contrast, body weight significantly decreased in both agomelatine‐treated groups, starting from day 7 and continuing across all time points. No significant differences were observed between the Ago‐20 and Ago‐40 groups. Values are expressed as means ± standard deviation. *Significant differences compared to the control group (*P <* 0.05).

**FIGURE 4:**
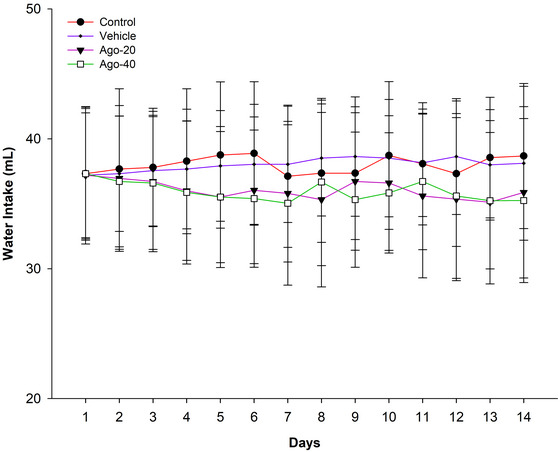
Water intake remained stable and comparable across all groups throughout the study period. No statistically significant differences were observed between the control, vehicle, Ago‐20 and Ago‐40 groups at any time point (*P >* 0.05). Values are expressed as means ± standard deviation.

During the experimental period, daily food intake (Figure 2) and body weight (Figure 3) were monitored across all groups. From days 1 to 14, the Ago‐20 and Ago‐40 groups exhibited significantly lower food intake compared to the control and vehicle groups (*P <* 0.05). On day 14, the mean daily food intake was significantly lower in the Ago‐20 group (20.14 ± 1.60 g) and Ago‐40 group (20.21 ± 1.32 g) compared to the control group (32.09 ± 1.58 g) and vehicle group (31.05 ± 1.92 g) (*P <* 0.05). However, no significant difference in food intake was observed between the Ago‐20 and Ago‐40 groups throughout the experiment (*P >* 0.05) (Figure 2). Changes in body weight followed a similar pattern. Starting from day 7, body weights in the Ago‐20 and Ago‐40 groups were significantly lower than those in the control and vehicle groups (*P <* 0.05). On day 14, body weight was significantly lower in the Ago‐20 (274.2 ± 6.3 g) and Ago‐40 (275.2 ± 7.2 g) groups compared to the control group (339.7 ± 8.3 g) and vehicle group (326.4 ± 7.76 g) (*P <* 0.05) (Figure 3). Similarly, no significant difference in body weight was detected between the Ago‐20 and Ago‐40 groups during the experimental period (*P >* 0.05). Water intake was monitored throughout the experimental period, and no statistically significant differences were observed among the groups at any time point (day 14 values: control: 38.68 ± 5.58 mL, vehicle: 38.12 ± 5.92 mL, Ago‐20: 35.88 ± 6.60 mL, Ago‐40: 35.25 ± 6.32 mL, *P* >  0.05) (Figure 4).

**FIGURE 5:**
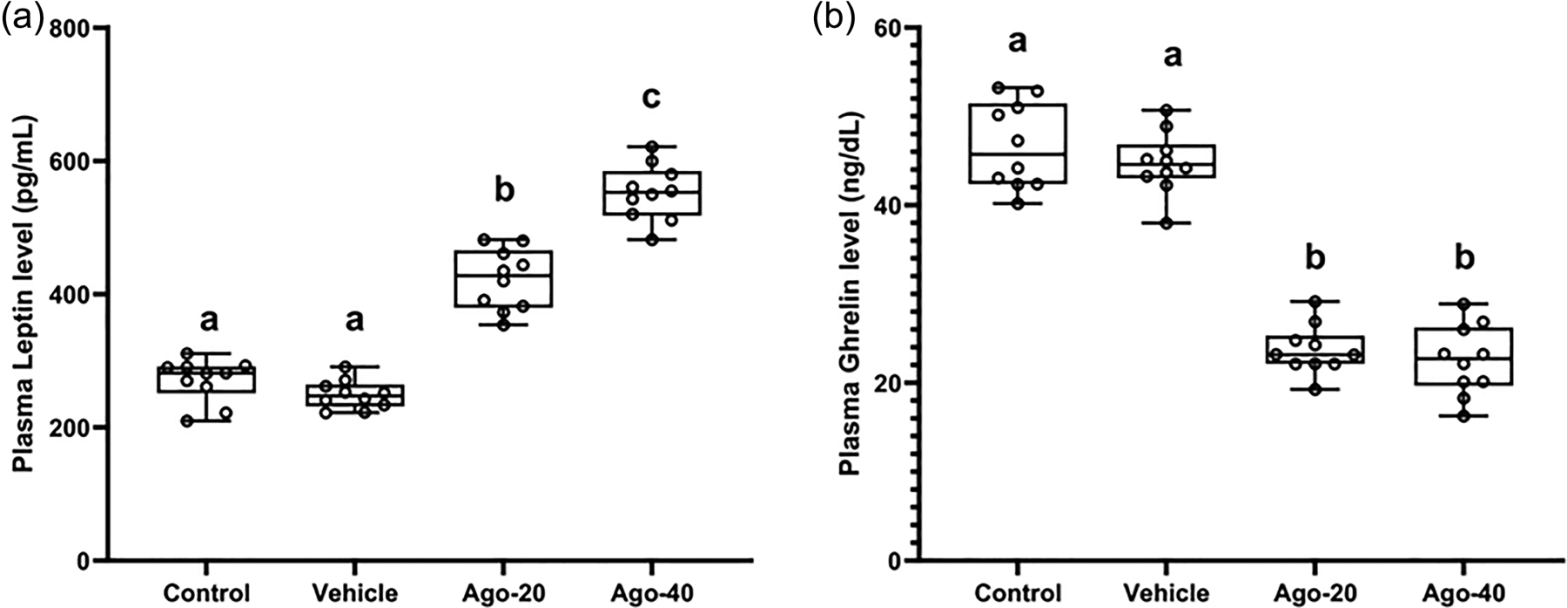
The effect of agomelatine administration on plasma leptin (a) and ghrelin (b) levels. The values are expressed as means ± standard deviation. Different superscript letters in the values indicate a significant difference between the groups (^a,b,c^ *P <* 0.05), while the same letters indicate no significant difference (*P >* 0.05).

Figure 5a shows the effects of agomelatine on plasma leptin levels, while Figure 5b shows its effects on plasma ghrelin levels. Comparisons between the experimental groups revealed that both 20 mg/kg (Ago‐20) and 40 mg/kg (Ago‐40) agomelatine treatments significantly increased plasma leptin levels compared to the control (271.10 ± 32.12 pg/mL) and vehicle groups (249.20 ± 21.52 pg/mL) (*P <* 0.05). Plasma leptin levels were significantly higher in the Ago‐20 group (422.20 ± 45.61 pg/mL) and Ago‐40 group (552.30 ± 41.67 pg/mL) compared to the control and vehicle groups (*P <* 0.05) (Figure 5a). In contrast, plasma ghrelin levels significantly decreased in both the Ago‐20 (23.73 ± 2.76 ng/dL) and Ago‐40 (22.54 ± 3.95 ng/dL) groups compared to the control (46.67 ± 4.84 ng/dL) and vehicle groups (44.74 ± 3.50 ng/dL) (*P <* 0.05) (Figure 5b).

**FIGURE 6:**
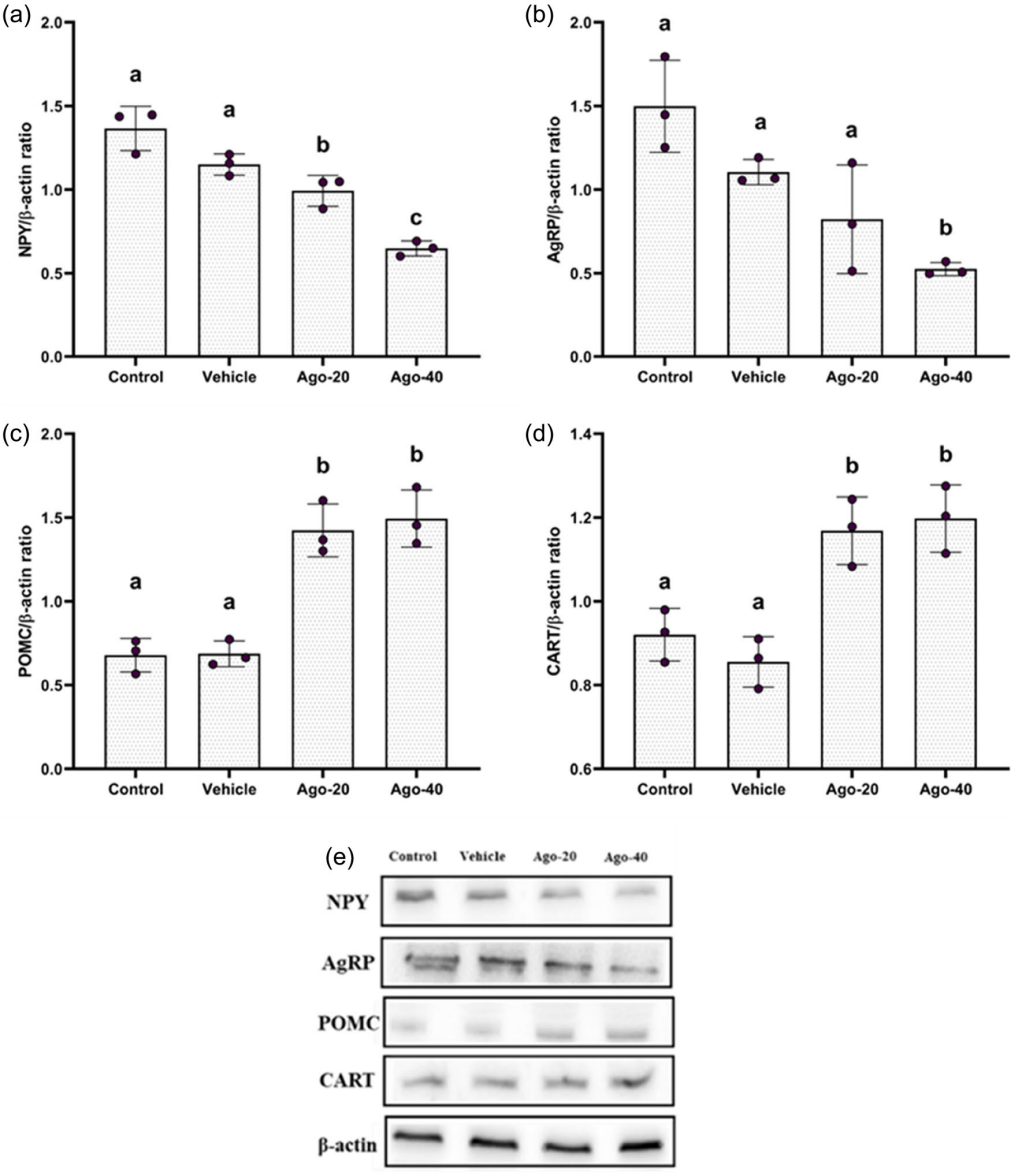
Densitometric analysis of hypothalamic NPY (a), AgRP (b), POMC (c), and CART (d) protein expression and the western blot gel images (e) in rats following agomelatine administration. The values are presented as means ± standard deviation. Different superscript letters in the values indicate significant differences between the groups (^a,b,c^ *P <* 0.05), while the same letters indicate no significant differences (*P >* 0.05).

The effects of agomelatine treatment on hypothalamic protein levels of NPY, AgRP, POMC and CART are shown in Figure 6a–d. NPY protein levels were significantly reduced in the Ago‐20 (0.99 ± 0.09) and Ago‐40 (0.61 ± 0.02) groups compared to the control (1.36 ± 0.13) and vehicle groups (1.15 ± 0.06) (*P <* 0.05; Figure 6a). AgRP protein levels were reduced as well, but a statistically significant reduction was only observed in the Ago‐40 group (0.52 ± 0.03) compared to the control (1.49 ± 0.27) and vehicle groups (1.10 ± 0.07) (*P <* 0.05; Figure 6b). POMC protein levels were significantly higher in the Ago‐20 (1.42 ± 0.15) and Ago‐40 (1.49 ± 0.17) groups compared to the control (0.67 ± 0.10) and vehicle groups (0.68 ± 0.07) (*P <* 0.05; Figure 6c). CART protein levels were significantly increased in the Ago‐20 (1.16 ± 0.08) and Ago‐40 (1.19 ± 0.08) groups compared to the control (0.92 ± 0.06) and vehicle groups (0.85 ± 0.06) (*P <* 0.05; Figure 6d).

## 4 DISCUSSION

Agomelatine acts as an agonist at melatonergic receptors (MT_1_/MT_2_) and as an antagonist at the 5‐HT_2C_ receptor, both of which play key roles in appetite regulation and body weight (Suriagandhi & Nachiappan, 2022; Yao et al., 2021). Preclinical studies have consistently demonstrated the weight‐reducing effects of melatonergic receptor activation (Guan et al., 2021). In this study, agomelatine was administered to healthy adult rats via oral gavage for 14 days, resulting in a decrease in food intake and body weight.

The findings of this study align with previous research that demonstrates the weight‐reducing effects of agomelatine in obese rats (Diez‐Echave et al., 2022; Promsan et al., 2022) and individuals with night eating syndrome (Milano et al., 2013; Zapp et al., 2017). Although the precise physiological mechanisms remain unclear, research on melatonin – a receptor analogue of agomelatine – provides valuable insights. For instance, melatonin administration has been shown to reduce food intake and body weight in healthy rats (Wang et al., 2021). Buonfiglio et al. reported that exogenous melatonin exerts anorexigenic effects by activating MT_1_ and MT_2_ receptors through hypothalamic neuropeptides (Buonfig et al., 2018). On the other hand, compounds that antagonize 5‐HT_2C_ receptors are often associated with increased appetite and weight gain (Yao et al., 2021). However, agomelatine acts as a neutral antagonist at 5‐HT_2C_ receptors, meaning it neither exhibits inverse agonism nor significantly alters receptor activity (Millan, 2022). Furthermore, while the combination of 5‐HT_2C_ receptor antagonism with histaminergic and/or muscarinic receptor antagonism typically heightens the risk of appetite stimulation and weight gain, agomelatine shows negligible affinity for these receptors (Di Giovanni & De Deurwaerdère, 2016; Millan, 2022). Notably, agomelatine's affinity for 5‐HT_2C_ receptors is approximately 1000 times weaker than its affinity for melatonergic receptors (Guardiola‐Lemaitre et al., 2014). Additionally, 5‐HT_2C_ receptors are mostly specific to the central nervous system and are not found in peripheral organs involved in appetite regulation (Bays, 2009). Based on this evidence, it can be hypothesized that the anorectic effects observed in this study are primarily mediated by agomelatine's melatonergic receptor activation.

MT_1_ and MT_2_ are known to be expressed in peripheral tissues such as adipose tissue and the stomach. These receptors play an important role in regulating appetite and energy homeostasis by controlling leptin secretion and modulating ghrelin production (Alonso‐Vale et al., 2008; Mustonen et al., 2001; Suriagandhi & Nachiappan, 2022). In this study, the increase in plasma leptin levels and the decrease in ghrelin levels after agomelatine administration suggest that agomelatine may also exert its effects through MT_1_/MT_2_ receptors in peripheral tissues. Leptin, a key anorexigenic hormone, plays a critical role in appetite regulation by acting on the hypothalamus (Timper & Brüning, 2017). It suppresses appetite through the activation of POMC/CART neurons and inhibition of AgRP/NPY neurons (Kristensen et al., 1998; Timper & Brüning, 2017). The significant increase in leptin levels observed after agomelatine treatment may explain the reductions in food intake and weight gain previously reported with leptin administration (Halaas et al., 1995). However, the exact mechanism underlying this increase in leptin levels following agomelatine treatment remains unclear. Interestingly, an increase in leptin levels following significant weight loss can be considered paradoxical (Obradovic et al., 2021). In this study, the rise in leptin levels may be linked to the activation of melatonergic receptors in tissues involved in leptin secretion. Furthermore, evidence suggests that melatonin may indirectly influence leptin production via its effects on insulin (Lee & Fried, 2009; Szewczyk‐Golec et al., 2015). Melatonin is proposed to activate intracellular signalling pathways similar to those of leptin by mimicking insulin's effects (Szewczyk‐Golec et al., 2015). In vitro studies by Alonso‐Vale et al. demonstrated that melatonin enhances leptin expression in adipocytes through insulin‐mediated mechanisms (Alonso‐Vale et al., 2006, 2005). Consistent with our findings, Song & Chen (2009) reported that melatonin supplementation increased plasma leptin levels in healthy rodents. However, in the in vitro phase of their study, melatonin alone, in the absence of insulin, did not alter leptin expression in adipocyte cells. Although insulin levels were not measured in this study, agomelatine may upregulate plasma leptin levels through pathways similar to insulin‐mediated mechanisms observed with melatonin. Further research is needed to validate this hypothesis. Ghrelin is described in the literature as a signal that triggers the initiation of feeding behaviour and modulates the firing rate of NPY/AgRP neurons in the hypothalamus (De Ambrogi et al., 2003). Studies in mice have demonstrated that exogenous ghrelin administration leads to increased food intake (Tschöp et al., 2000). In line with our findings, both preclinical and clinical research have reported that melatonergic activation reduces plasma ghrelin levels (Celinski et al., 2009; Mustonen et al., 2001).

Furthermore, in the present study, the increase in the protein expression of anorexigenic neuropeptides such as POMC and CART, along with the decrease in the protein expression of orexigenic neuropeptides such as AgRP and NPY in the hypothalamus following agomelatine administration, suggests that agomelatine may have a direct effect on the feeding centres in the hypothalamus. The current literature lacks studies specifically investigating the relationship between agomelatine and neuropeptides (NPY, AgRP, CART and POMC) involved in appetite regulation in healthy rats. However, evidence suggests that MT_1_ and MT_2_ receptor signalling in healthy rats contributes to the central regulation of appetite, resulting in reduced food intake and body weight (Buonfig et al., 2018). Indeed, the ARN, which plays a central role in appetite regulation, exhibits moderate immunoreactivity for MT1 and MT2 receptors (Ng et al., 2017; Wu et al., 2006). Evidence suggests that MT_1_ and MT_2_ agonism may modulate appetite metabolism in the ARN by influencing anorexigenic POMC neurons as well as orexigenic NPY and AgRP neurons (Suriagandhi & Nachiappan, 2022). Consistent with these findings, disrupted MT_1_ signalling in ARN has been associated with increased food intake, indicating that MT_1_ signalling is essential for maintaining proper appetite regulation in the ARN (Buonfig et al., 2019). Additionally, Fischer et al. (2017) reported that MT_1_ receptor knockout in mice led to reduced POMC expression in the ARN. In accordance with these findings, melatonin administration in high‐fat diet‐fed obese mice and *Danio rerio* reduced NPY mRNA expression (Montalbano et al., 2018; Ríos‐Lugo et al., 2015). In the present study, agomelatine administration decreased NPY/AgRP protein levels while increasing POMC/CART protein levels. These findings are consistent with previous research indicating that melatonergic receptor agonism plays a role in the central regulation of appetite, further supporting the involvement of agomelatine in the modulation of feeding behaviour.

On the other hand, activation of the melatonergic receptors MT_1_ and MT_2_ has also been associated with effects such as the regulation of the sleep–wake cycle and reduction of locomotor activity. In this context, it is theoretically plausible that the potential sedative or hypnotic effects of agomelatine may contribute to its impact on appetite and body weight (Liu et al., 2016; Marseglia et al., 2015). However, current literature indicates that agomelatine administration, including doses comparable to those used in our study, does not lead to significant changes in basal locomotor activity in healthy rats (Barbosa‐Méndez et al., 2023; Tchekalarova et al., 2017). Additionally, the observation that water intake remained similar across all groups in our study is noteworthy. This may be considered strong evidence suggesting that locomotor activity was not affected, as previous studies have reported parallel changes between locomotor activity and water consumption (Bannai et al., 2003; De Santis et al., 2014). Furthermore, Mairesse et al. (2013) reported that agomelatine treatment did not alter sleep–wake architecture in healthy rats. Moreover, clinical studies have shown that agomelatine restores the sleep–wake rhythm in patients with depression without inducing side effects such as sedation (Grosshans et al., 2014; Kennedy & Eisfeld, 2007; Rouillon, 2006). Taken together, these findings suggest that the effects observed in our study on appetite and body weight may stem from the direct metabolic and neuropeptidergic actions of agomelatine, rather than secondary effects related to general behavioural activity or sleep–wake regulation. Nonetheless, to completely rule out potential sedative and hypnotic influences, further studies are needed to comprehensively investigate the effects of agomelatine on locomotor activity and behavioural parameters.

### 4.1 Conclusion

This study demonstrates that agomelatine reduces food intake and body weight. Specifically, agomelatine treatment increased plasma leptin levels and decreased ghrelin levels in both experimental groups. In the hypothalamus, agomelatine treatment led to significant increases in POMC and CART protein levels, while NPY and AgRP protein levels were significantly decreased. These findings suggest that agomelatine may be a potential therapeutic option for the management of obesity and metabolic disorders; however, certain limitations should be noted:

- It remains unclear whether the observed changes in hypothalamic neuropeptide levels are a direct effect of agomelatine or the result of indirect metabolic adaptations (such as increased leptin or decreased ghrelin levels).
- Receptor antagonists were not used to determine the specific effects of agomelatine on feeding behaviour.
- Key parameters related to glucose and energy metabolism were not measured.
- The treatment duration was limited to 14 days. It is unknown whether the effects of agomelatine would persist with long‐term use or be reversible upon treatment discontinuation. Future studies should include extended treatment periods and post‐treatment follow‐up.

In conclusion, this study provides important preliminary findings on the effects of agomelatine administration on feeding behaviour. However, to better understand the underlying mechanisms and confirm its therapeutic potential, longer‐term, comprehensive and mechanistic studies are needed.

## AUTHOR CONTRIBUTIONS

Conception or design of the work: Suat Tekin, Engin Korkmaz. Acquisition or analysis or interpretation of data for the work: Suat Tekin, Engin Korkmaz, Yavuz Erden, Çiğdem Tekin. Drafting the work: Engin Korkmaz. Revising it critically for important intellectual content: Suat Tekin, Yavuz Erden, Çiğdem Tekin. All authors have read and approved the final version of the manuscript, agree to be accountable for all aspects of the work in ensuring that questions related to the accuracy or integrity of any part of the work are appropriately investigated and resolved, and confirm that all persons designated as authors qualify for authorship and that all those who qualify for authorship are listed.

## CONFLICT OF INTEREST

The authors declare they have no conflicts of interest.

## Data Availability

The data that support the findings of this study are available from the corresponding author upon reasonable request.
